# RBM15 promotes hypoxia/reoxygenation-induced ferroptosis in human cardiomyocytes by mediating m6A modification of ACSL4

**DOI:** 10.1186/s41065-025-00453-0

**Published:** 2025-07-18

**Authors:** Yi Cheng, Jiamin Wan, Yingyue Xu, Shasha Liu, Linfeng Li, Jing Zhou, Fuyan Xie

**Affiliations:** 1 Department of Geriatric Medicine, Bishan Hospital of Chongqing, Bishan Hospital of Chongqing Medical University, No. 9, Double Star Avenue, Biquan Street, Bishan District, Chongqing, 402760 China; 2 Department of Emergency Medicine, Bishan Hospital of Chongqing, Bishan hospital of Chongqing Medical University, Chongqing, 402760 China

**Keywords:** AMI, RBM15, ACSL4, AC16, m6A

## Abstract

**Background:**

Acute myocardial infarction (AMI) refers to the acute necrosis of part of the myocardium caused by persistent and severe myocardial ischemia. The aim of the study was to investigate the effect of RNA binding motif protein 15 (RBM15) and acyl-CoA synthetase long chain family member 4 (ACSL4) on ischemia/reperfusion (I/R)-induced ferroptosis of cardiomyocytes.

**Methods and results:**

AC16 cells were treated with hypoxia/reoxygenation (H/R) to establish an in vitro myocardial infarction cell model. Quantitative real-time polymerase chain reaction (qRT-PCR) and western blot assay were used to determine gene expression. Cell Counting Kit-8 (CCK-8) assay was conducted to investigate cell viability. Ferroptosis level was evaluated by commercial kits. N6-methyladenosine (m6A) level was examined by M6A quantification analysis. RNA immunoprecipitation (RIP) assay, methylated RNA Immunoprecipitation (meRIP) assay and dual-luciferase reporter assay were adopted to verify the combination between RBM15 and ACSL4. ACSL4 mRNA stability was analyzed by Actinomycin D treatment. RBM15 mRNA level was increased in AMI patients’ serums and H/R-induced AC16 cells. Silencing of RBM15 promoted H/R-mediated AC16 cell viability and inhibited H/R-induced AC16 cell oxidative stress and ferroptosis. Moreover, it was demonstrated that RBM15 knockdown inhibited m6A modification of ACSL4 and suppressed the stability of ACSL4 mRNA. Furthermore, ACSL4 overexpression restored the effects of RNM15 silencing on H/R-induced AC16 cell oxidative injury and ferroptosis.

**Conclusion:**

RBM15 silencing repressed H/R-induced ferroptosis in human cardiomyocytes through regulating m6A modification of ACSL4.

**Supplementary Information:**

The online version contains supplementary material available at 10.1186/s41065-025-00453-0.

## Introduction

Acute myocardial infarction (AMI) is a disease due to acute coronary artery blockages in the corresponding myocardial regional problems, leading to myocardial necrosis [1, 2]. Timely restoration of ischemic myocardial perfusion is the most effective strategy for the treatment of AMI, which can significantly reduce morbidity and mortality [3, 4]. However, after the restore blood flow and reperfusion, myocardial injury is aggravated and cardiomyocyte apoptosis is induced, a process called myocardial ischemia-reperfusion (I/R) injury [5, 6]. Exploring the underlying mechanisms of I/R-induced myocardial injury is crucial for AMI therapy.

N6-methyladenosine (m6A) is one of the most common and abundant types of internal decoration in eukaryotic RNAs [7]. Research shows that m6A plays an important role in the occurrence and development in a variety of diseases, such as cardiovascular disease, diabetes and cancers [8–10]. RNA binding motif protein 15 (RBM15) is an m6A methylation regulatory protein and has been reported to be a vital regulator in the progression of human disorders [11, 12]. Moreover, RBM15 was found to be a new biomarker of myocardial infarction [13, 14]. However, the exact roles and underlying mechanisms of RBM15 in AMI development have not been investigated.

Acyl-CoA synthetase long chain family member 4 (ACSL4) is an important isoenzyme in the metabolism of polyunsaturated fatty acids (PUFAs), which can enhance the production of lipid peroxides and promote ferroptosis [15, 16]. Cui et al. verified that ACSL4 was able to aggravate the ischemic injury of brain [17]. Yu et al. indicated that ACSL4 participated in the regulation of ferroptosis and myocardial ischemia injury through epigallocatechin gallate (EGCG)/miR-450b-5p/ACSL4 axis [18]. In this study, RBM15 was found to mediate m6A modification of ACSL4. Nevertheless, the relation between RBM15 and ACSL4 in regulating AMI development is not reported.

In the present research, human cardiomyocyte cells were exposed to hypoxia/reoxygenation (H/R) to mimic the myocardial I/R injury. Moreover, the exact roles and mechanisms of RBM15 and ACSL4 in H/R-induced cell damage were investigated.

## Materials and methods

### Collection of serum samples

65 AMI patients at Bishan Hospital of Chongqing, Bishan Hospital of Chongqing Medical University were enrolled in this study. 65 healthy individuals were recruited as controls. The bloods were harvested from the participants and centrifuged to obtain serums. The study was authorized by the Ethics Committee of Bishan Hospital of Chongqing, Bishan Hospital of Chongqing Medical University and written informed consents were signed by all participants.

### Measurements of cardiac troponin I (cTnI) and creatine kinase MB (CK-MB)

The concentrations of cTnI and CK-MB in the serums of AMI patients were examined with human cTnI ELISA kit and human CK-MB ELISA kit (Abcam, Cambridge, MA, USA), respectively.

### Cell culture and hypoxia/reoxygenation (H/R) treatment

Human cardiomyocyte cell line AC16 was purchased from Procell (Wuhan, China). The cells were cultured in the specific culture medium of DMEM/F12 (containing HEPES) + 10% fetal bovine serum (FBS) + 1% penicillin-streptomycin (Procell) in a humid incubator containing 5% CO_2_ at 37 °C.

To construct the cell model of myocardial H/R injury, AC16 cells were maintained for 16 h at 37 °C in a hypoxic condition of 1% O_2_, 5% CO_2_ and 94% N_2_ and then reoxygenated for 6 h in a condition of 20% O_2_, 5% CO_2_ and 75% N_2_ [19–21]. In the control group, AC16 cells were cultured at 37 °C in the normal incubator of 5% CO_2_ and 95% air [22].

### Cell transfection

The small interfering RNA (si-RNA) against RBM15 (si-RBM15) or ACSL4 (si-ACSL4) was synthesized for the knockdown of RBM15 or ACSL4 and si-NC was the control. RBM15 overexpression vector (RBM15) was constructed to upregulate RBM15 expression and empty (vector) was used as relative control. To overexpress ACSL4, ACSL4 overexpression vector (pcDNA-ACSL4) was generated, and the related empty vector (pcDNA-NC) was used as control. The synthetic oligonucleotides or vectors were offered by GeneCopoeia (Guangzhou, China). For cell transfection, Lipofectamine 2000 (Invitrogen, Carlsbad, CA, USA) was adopted as the described in the instructions.

### Cell counting Kit-8 (CCK-8) assay

AC16 cells were seeded in the 96-well plates for 24 h and then 10 µL CCK-8 (Beyotime, Shanghai, China) was added into the well for 2 h. The absorbance at 450 nm was determined by a microplate reader.

### Measurement of lactate dehydrogenase (LDH), reactive oxygen species (ROS), malondialdehyde (MDA), superoxide dismutase (SOD) and Fe^2+^

LDH level in AC16 cells was measured using Promega CytoTox 96^®^ kit (Promega, Madison, WI, USA) in line with the protocols.

The level of ROS in AC16 cells was determined with ROS assay kit (Abcam) according to the manufacturers’ instructions. In brief, the treated AC16 cells were kept with ROS working solution for 45 min in a dark atmosphere and the fluorescence intensity was examined utilizing a flow cytometer.

To examine MDA accumulation, SOD activity and Fe^2+^ level, MDA assay kit (Nanjing Jiancheng Bioengineering Institute, Nanjing, China), SOD assay kit (Nanjing Jiancheng Bioengineering Institute) and iron assay kit (Abcam) were used strictly in line with the protocols.

### Quantitative real-time polymerase chain reaction (qRT-PCR)

The RNAs in serums and cells were extracted using TRIzol reagent (Invitrogen). The RNAs were subjected to M-MLV Reverse Transcriptase Kit (Promega) for the synthesis of DNAs. Next, qRT-PCR assay was conducted using BeyoFast™ SYBR Green qPCR Mix (Beyotime) on the ABI 7500 PCR system. mRNA expression was determined through 2^−ΔΔCt^ method and β-actin served as the internal reference. Table 1 exhibited the used primers.

**Table 1:** Primer sequences for qRT-PCR

Gene	Forward (5’-3’)	Reverse (5’-3’)
RBM15	ATGCCTTCCCACCTTGTGAG	GGTCAGCGCCAAGTTTTCTC
ACSL4	GAAAAAGAGGACATTTAAAAACGCT	CAAGGCTGTCCTTCTTCCCA
β-actin	CTTCGCGGGCGACGAT	CCACATAGGAATCCTTCTGACC

### Western blot

AC16 cells were lysed in RIPA buffer (Beyotime) to extract the proteins, which were then quantified using BCA protein assay kit (Tiangen, Beijing, China). The proteins were separated by the SDS-PAGE electrophoresis and blotted on PVDF membranes. Next, the proteins were blocked in 5% skim milk for 4 h, incubated overnight with the primary antibody against RBM15 (ab300467; Abcam), glutathione peroxidase 4 (GPX4; ab41787; Abcam), ACSL4 (ab227256; Abcam), ferritin heavy chain 1 (FTH1; ab65080; Abcam), nuclear receptor coactivator 4 (NCOA4; ab86707; Abcam) or β-actin (ab5694; Abcam) and maintained with HRP-linked goat-anti-rabbit secondary antibody (ab6721; Abcam) for 2 h. At last, the proteins were visualized by enhanced chemiluminescence (ECL) kit (Beyotime).

### Detection of glutathione (GSH) and GSSG levels

GSH assay kit and GSSG assay kit were purchased from Nanjing Jiancheng Bioengineering Institute to detect the levels of GSH and GSSG in AC16 cells according to the manufacturers’ instructions.

### RNA Immunoprecipitation (RIP) assay

The Magna RIP™ RNA Binding Protein Immunoprecipitation Kit (Millipore, Bedford, MA, USA) was used for RIP assay. Briefly, AC16 cells were lysed in RIP lysis buffer and cell lysis was incubated with magnetic beads conjugated with antibody against Ago2 or IgG for 6 h. Then the samples were washed and the immunoprecipitated RNA was extracted from the beads and subjected to qRT-PCR for ACSL4 enrichment.

### M6A quantification analysis

After relevant treatment and transfection, AC16 cells were seeded into 96-well plates and then total RNA was extracted. Then the RNAs were used to determine the level of m6A in AC16 cells with m6A RNA methylation quantification kit (Abcam). In brief, 200 ng RNAs were added to the test wells, and capture and assay antibody solutions were added to each well. The m6A level was determined by measuring the OD value at 450 nm.

### Methylated RNA Immunoprecipitation (meRIP) assay

Protein A/G beads were incubated with anti-IgG antibody (Abcam) or anti-m6A antibody (Abcam) in the IP buffer (2 mM EDTA, 1% NP-40, 140 mM NaCl, and 20 mM Tris) for 1 h. The RNAs in si-NC, si-RBM15, vector or RNM15 transfected AC16 cells were extracted and incubated overnight in the IP buffer together with the bead-antibody complex. Subsequently, the RNAs bounds to the beads were extracted and subjected to qRT-PCR for ACSL4 enrichment.

### Dual-luciferase reporter assay

The wild-type (WT) or mutant-type (MUT) ACSL4 fragments containing or lacking the m6A modification sites of RBM15 were inserted into pGL3 reporter gene vector (Promega). The constructed vector (WT-ACSL4/MUT-ACSL4) was transfected into AC16 cells together with si-NC/si-RBM15. After 48 h, the luciferase intensity was examined with a Dual-Luciferase Reporter Assay Reagent (Promega).

### Actinomycin D treatment

This assay was conducted to evaluate the stability of RBM15 mRNA stability. In brief, the si-NC, si-RBM15, vector or RBM15 transfected AC16 cells were treated with Actinomycin D (Sigma-Aldrich) at 0 h, 2 h, 4 h and 6 h. Subsequently, RBM15 mRNA level was examined by qRT-PCR.

### Establishment of AMI mice model

The AMI mice model was established by ligation of LAD artery. The anesthetic drug was isoflurane and the airflow rate was 0.8–1.0 L /min. The mice were divided into 3 groups: Sham, MI, MI + sh-RBM15. After disinfecting the surgical area, a 1–2 cm skin incision was made between the 3rd and 4th ribs of the left chest to expose the heart. The pericardium was separated, the heart was exteriorized, and AMl was induced by rapid ligation of the LAD coronary artery with 6.0 prolene sutures at a distance of approximately 1 mm from the left atrial appendage, 2 mm in width and depth. Successful occlusion of the LAD coronary artery was confirmed by ECG with st segment elevation. Ligation was completed and the heart was immediately placed back into the thoracic cavity, which was then closed.

In MI + sh-RBM15 group, the mice were transfected with sh-RBM15 transfected AC16 cells and the other steps were in line the MI group.

The Sham group underwent the same operation without ligation of the left anterior descending artery. Anesthesia with 5% isoflurane was administered after ligation. Myocardial tissues from the left ventricular anterior wall were immediately harvested for analysis.

### Statistics analysis

The data in this study were harvested from three independent experiments. The data were analyzed by GraphPad Prism 7 software and exhibited as mean ± SD. The linear correlation between RBM15 level and cTnI/CK-MB level in AMI patients was analyzed by Spearman’s correlation coefficient analysis. The differences between two groups were analyzed by Student’s *t*-test and the differences among three groups were analyzed by one-way ANOVA. *P* value less than 0.05 was considered as significant.

## Results

### RBM15 was upregulated in AMI patients’ serums

**Fig. 1:**
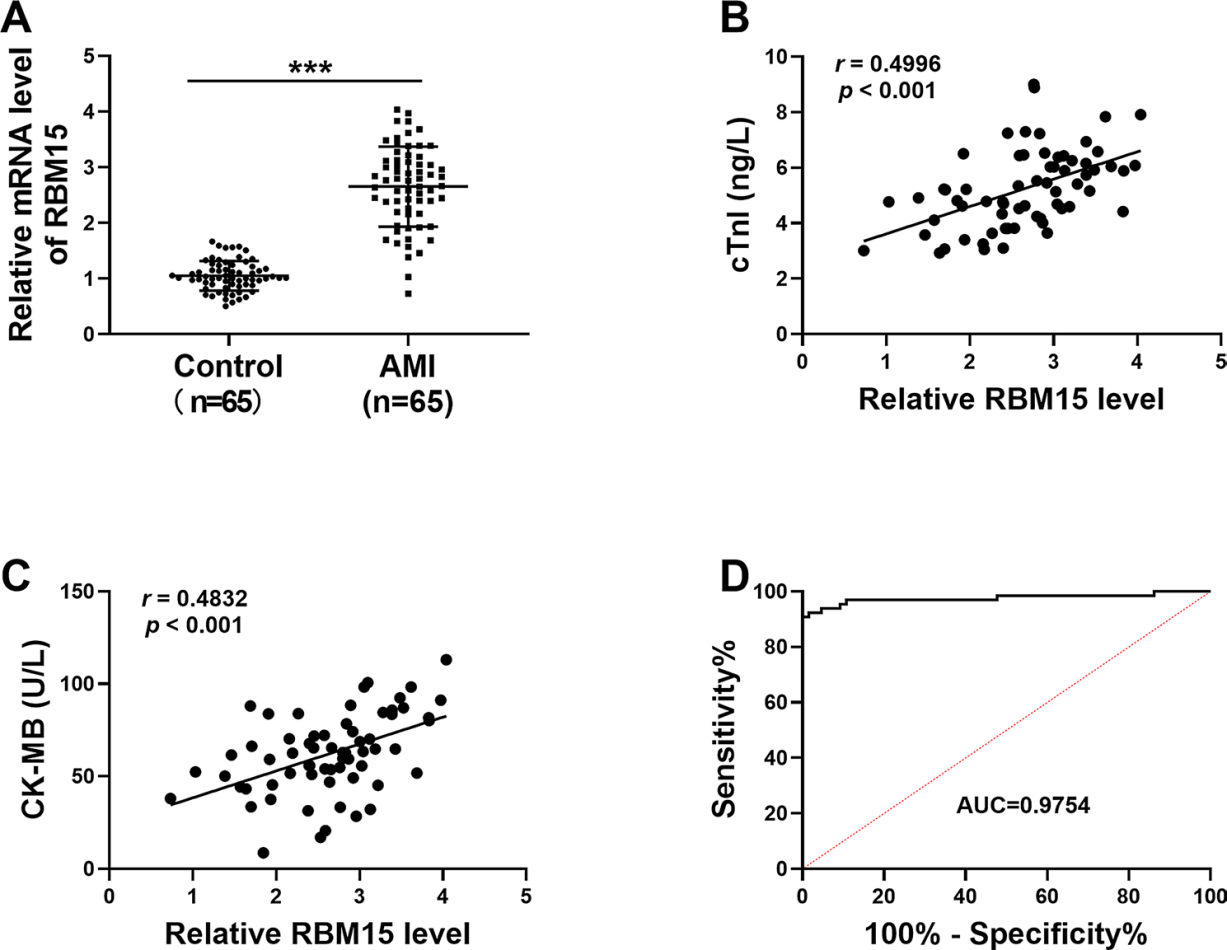
High level of RBM15 in the serums of AMI patients. (**A**) The mRNA level of RBM15 in the serums of AMI patients and healthy controls was determined by qRT-PCR. (**B** and **C**) The linear correlation between RBM15 level and cTnI/CK-MB level in AMI patients was analyzed by Spearman’s correlation coefficient analysis. (**D**) Diagnostic value of RBM15 for AMI was estimated. ***P < 0.001

qRT-PCR assay was conducted to determine the expression of RBM15 in the serums of AMI patients (*n* = 55) and healthy volunteers (*n* = 50). The results showed that RBM15 mRNA level was upregulated in AMI patients’ serums compared to control serums (Fig. 1A). Spearman’s correlation coefficient analysis was conducted to estimate the correlation between the levels of RBM15 and myocardial injury markers (cTnI and CK-MB) in the serums of AMI patients. The results showed that RBM15 level in AMI patients was positively correlated with cTnI and CK-MB levels (Fig. 1B and C). ROC analysis showed that the AUC was 0.9754, indicating the good diagnostic value of RBM15 for AMI (Fig. 1D).

### RBM15 knockdown inhibited H/R-induced oxidative injury in AC16 cells

**Fig. 2:**
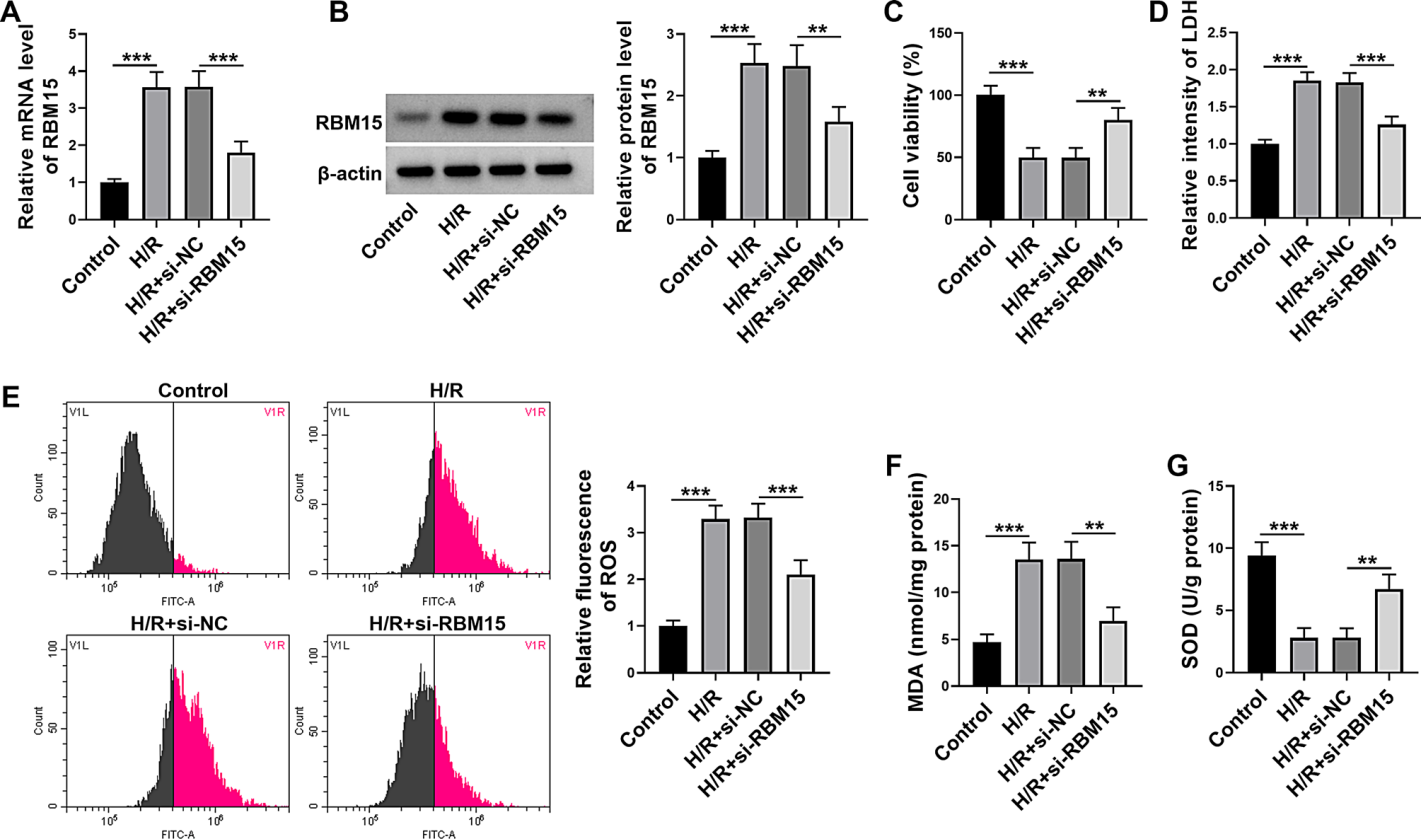
Deficiency of RBM15 ameliorated H/R-stimulated oxidative damage in AC16 cells. AC16 cells were treated with H/R, H/R + si-NC or H/R + si-RBM15 and untreated cells were control. (**A** and **B**) The mRNA and protein levels in AC16 cells were measured by qRT-PCR assay and western blot assay, respectively. (**C**) AC16 cell viability was explored by CCK-8 assay. (**D**-**G**) The levels of LDH, ROS, MDA and SOD in AC16 cells were examined by related commercial kits. **P < 0.01, ***P < 0.001

To explore the function of RBM15 on H/R-induced oxidative injury in AC16 cells, AC6 cells were transfected with si-RBM15 or si-NC and then subjected to H/R treatment. As exhibited in Fig. 2A and B, H/R treatment markedly elevated the mRNA and protein levels of RBM15 in AC16 cells, and si-RBM15 transfection reduced the mRNA and protein levels of RBM15 in H/R-treated AC16 cells. CCK-8 assay indicated that H/R treatment suppressed AC16 cell viability, while RBM15 knockdown alleviated the effect (Fig. 2C). Furthermore, H/R treatment enhanced LDH intensity, ROS generation and MDA content and reduced SOD activity in AC16 cells, while these effects mediated by H/R treatment were reversed by silencing RBM15 (Fig. 2D-G). These results indicated that RBM15 knockdown repressed H/R-induced oxidative damage in AC16 cells.

### Silencing of RBM15 inhibited H/R-induced ferroptosis in AC16 cells

**Fig. 3:**
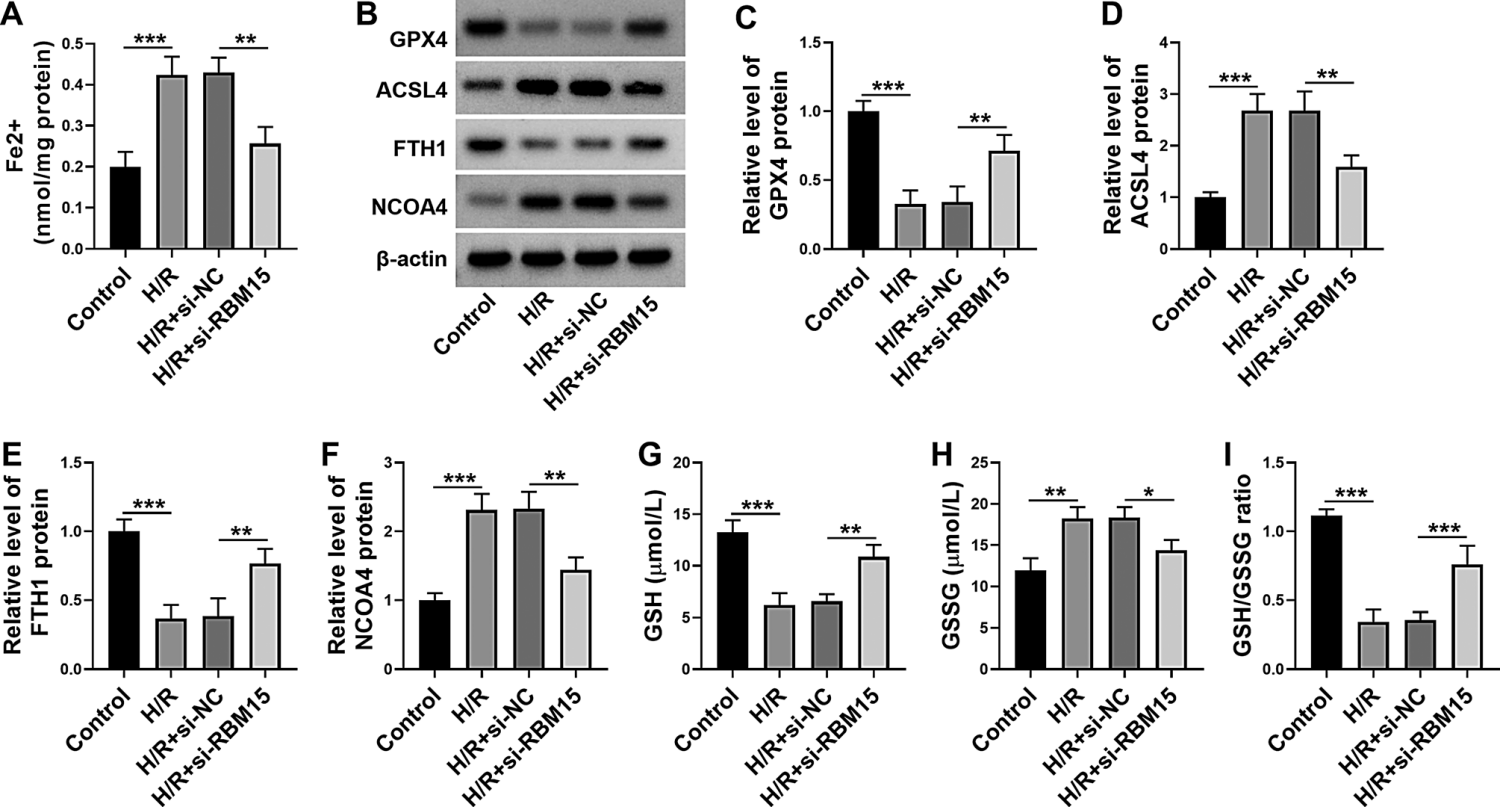
RBM15 knockdown repressed H/R-stimulated ferroptosis in AC16 cells. AC16 cells were divided into 4 groups: control, H/R, H/R + si-NC and H/R + si-RBM15. (**A**) Fe^2+^ level in AC16 cells was examined by indicated commercial kit. (**B**-**F**) The protein levels of GPX4, ACSL4, FTH1 and NCOA4 in AC16 cells were measured by western blot. (**G**-**I**) The levels of GSH, GSSG and GSH/GSSG in AC16 cells were examined with indicated commercial kits. *P < 0.05, **P < 0.01, ***P < 0.001

H/R treatment led to a significant upregulation of Fe^2+^ in AC16 cells, whereas RBM15 silencing restored the effect (Fig. 3A). Next, western blot assay was conducted to determine the expression of ferroptosis-related markers, including GPX4, ACSL4, FTH1 and NCOA4. As a result, H/R treatment decreased the protein levels of GPX4 and FTH1 and increased the protein levels of ACSL4 and NCOA4 in AC16 cells, while these effects were ameliorated by RBM15 downregulation (Fig. 3B-F). In addition, the content of anti-oxidant GSH was reduced and the content of GSSG was elevated in AC16 cells following H/R treatment, with RBM15 knockdown abated the effects (Fig. 3G and H). The ration of GSH/GSSG in AC16 cells was declined after H/R stimulation, but the effect was reversed by downregulating RBM15 (Fig. 3I). Taken together, RBM15 knockdown inhibited H/R-induced ferroptosis in AC16 cells.

### RBM15 mediated m6A modification of ACSL4

**Fig. 4:**
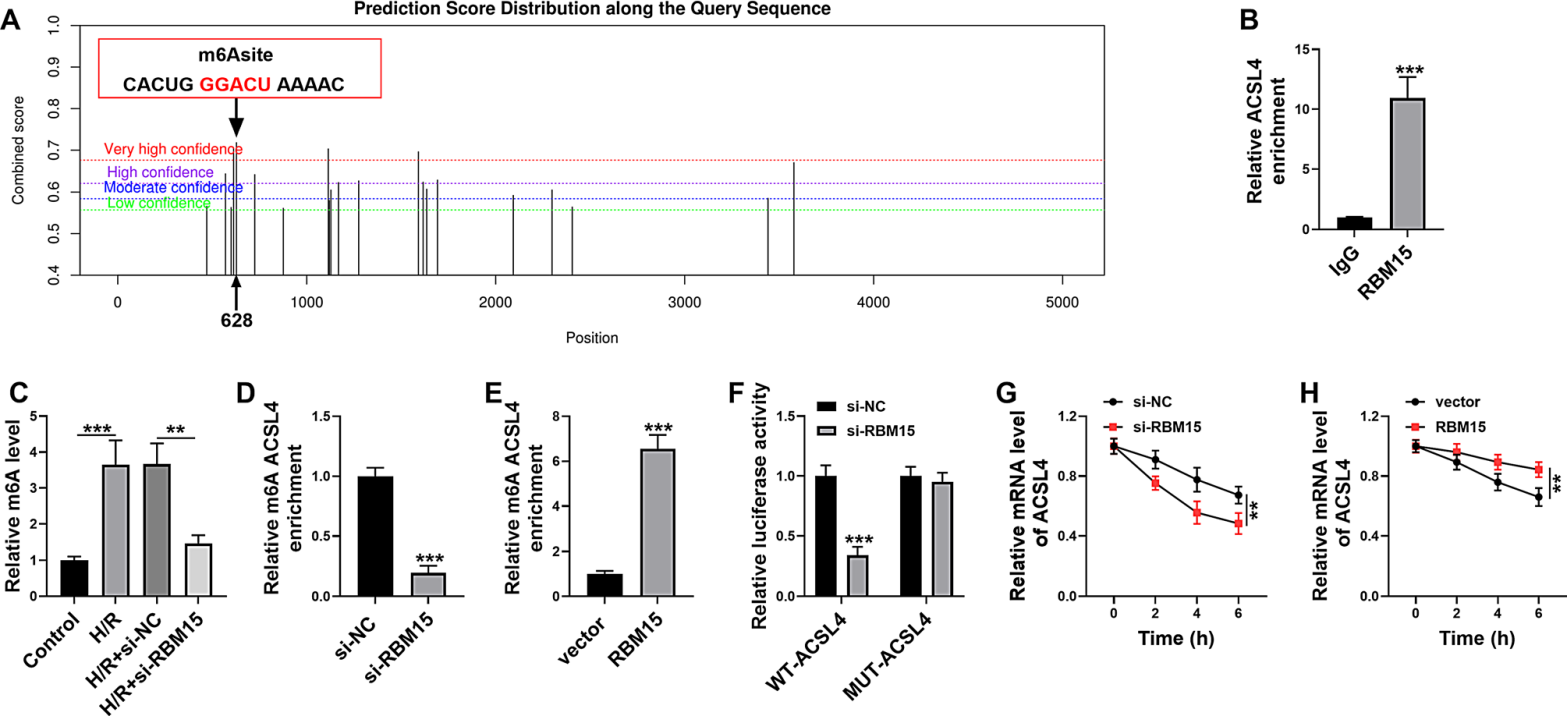
RBM15 regulated ACSL4 expression through m6A modification. (**A**) SRAMP database showed that RBM15 contained the m6A modification sites of ACSL4. (**B**) The combination between RBM15 and ACSL4 was verified by RIP assay. (**C**) m6A levels in AC16 cells in control, H/R, H/R + si-NC and H/R + si-RBM15 controls were determined by m6A quantification assay kit. (**D**-**F**) The interaction between RBM15 and ACSL4 was verified by MeRIP assay and dual-luciferase reporter assay. (**G** and **H**) After Actinomycin D treatment for 0 h, 2 h. 4 h and 6 h, ACSL4 mRNA level in AC16 cells was determined by qRT-PCR. **P < 0.01, ***P < 0.001

Subsequently, the underlying mechanism of RBM15 in regulating H/R-induced ferroptosis was explored. SRAMP database showed that RBM15 contained the m6A modification sites of ACSL4 (Fig. 4A). RIP assay demonstrated that ACSL4 could bind to RBM15 (Fig. 4B). Then m6A level was determined and we found that m6A was increased in AC16 cells after H/R treatment and the effect was abolished by RBM15 knockdown (Fig. 4C). MeRIP assay showed that m6A-modified ACSL4 level was reduced in si-RBM15-transfected AC16 cells and elevated in RBM15-transfected AC16 cells (Fig. 4D and E). Dual-luciferase reporter assay showed that RBM15 knockdown repressed the luciferase activity of WT-ACSL4 in AC16 cells, but had no effect on the luciferase activity of MUT-ACSL4 (Fig. 4F). These findings demonstrated the combination between RBM15 and ACSL4. Actinomycin D assay was then conducted to estimate the effect of RBM15 on the stability of ACSL4 mRNA. As a result, RBM15 knockdown inhibited ACSL4 mRNA stability and RBM15 overexpression enhanced ACSL4 mRNA stability in AC16 cells (Fig. 4G and H). These results illustrated that RBM15 mediated m6A modification of ACSL4 and regulated its expression.

### Knockdown of ACSL4 repressed H/R-induced ferroptosis in AC16 cells

To explore the function of ACSL4 in ferroptosis in AC16 cells, AC16 cells were transfected with si-ACSL4 to knock down ACSL4 expression and the transfection efficiency was estimated by western blot (Figure S1A). ACSL4 deficiency reduced Fe^2+^ level in H/R-induced AC16 cells (Figure S2B). Moreover, it was demonstrated that H/R-mediated effects on GSH content, GSSG content and GSH/GSSG ratio in AC16 cells were all alleviated by downregulating ACSL4 (Figure S2C-E). These outcomes illustrated that ACSL4 silencing restrained H/R-stimulated ferroptosis in AC16 cells.

### Overexpression of ACSL4 restored the effect of RBM15 knockdown on H/R-induced AC16 cell damage

**Fig. 5:**
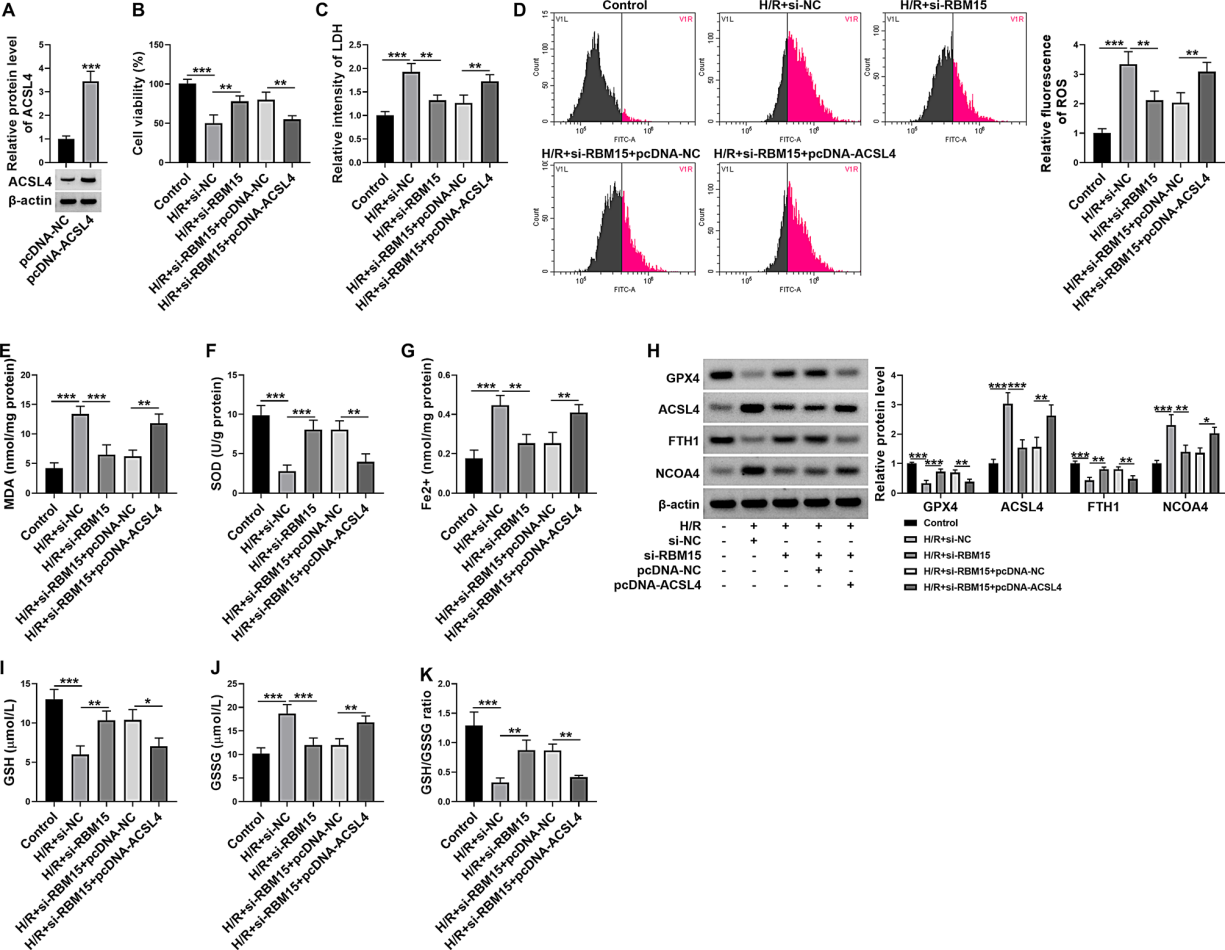
RBM15 knockdown repressed H/R-induced AC16 cell damage by altering ACSL4 expression. (**A**) The protein level of ACSL4 in AC16 cells transfected with pcDNA-NC or pcDNA-ACSL4 was measured by western blot. (**B**-**K**) AC16 cells were divided into 5 groups: control, H/R + si-NC, H/R + si-RBM15, H/R + si-RBM15 + pcDNA-NC and H/R + si-RBM15 + pcDNA-ACSL4. (**B**) AC16 cell viability was assessed by CCK-8 assay. (**C**-**G**) The levels of LDH, ROS, MDA, SOD and Fe^2+^ in AC16 cells were examined by commercial kits. (**H**) The protein levels of GPX4, ACSL4, FTH1 and NCOA4 in AC16 cells were measured by western blot. (**I**-**K**) The levels of GSH, GSSG and GSH/GSSG in AC16 cells were examined by the indicated kits. *P < 0.05, **P < 0.01, ***P < 0.001

As presented in Fig. 5A, the transfection of ACSL4 overexpression vector (pcDNA-ACSL4) led to the upregulation of ACSL4 protein level in AC16 cells in comparison with pcDNA-NC control group. As displayed by CCK-8 assay, RBM15 silencing facilitated H/R-treated AC16 cell viability, with ACSL4 enhancement ameliorated the effect (Fig. 5B). Deficiency of RBM15 reduced LDH level, ROS generation, MDA content and Fe^2+^ level and elevated SOD activity in H/R-induced AC16 cells, while the effects were ameliorated by upregulating ACSL4 (Fig. 5C-G). Western blot assay showed that RBM15 knockdown increased GPX4 and FTH1 protein levels and decreased ACSL4 and NCOA4 protein levels in H/R-treated AC16 cells, while ACSL4 overexpression abated the effects (Fig. 5H). In addition, GSH level and GSH/GSSG ration were elevated and GSSG level was reduced in si-RBM15-transfected H/R-treated AC16 cells, whereas ACSL4 overexpression alleviated the effects (Fig. 5I-K). Collectively, RBM15 knockdown alleviated H/R-induced AC16 cell oxidative stress and ferroptosis by m6A modification of ACSL4.

### RBM15 stabilized and regulated ACSL4 to promote H/R-induced AC16 cell ferroptosis

**Fig. 6:**
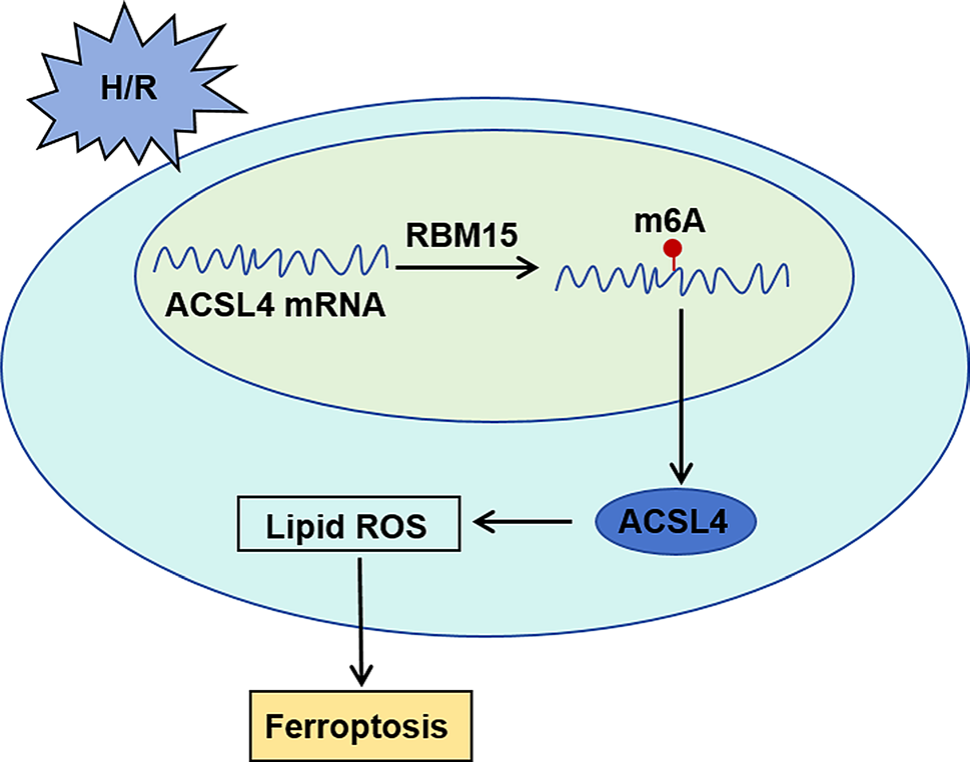
The abridged general view of RBM15/ACSL4 axis in regulating H/R-induced AC16 cell injury

In conclusion, RBM15 modulated ACSL4 expression through m6A modification, thereby promoting H/R-induced AC16 cell oxidative stress and ferroptosis (Fig. 6).

### Function of RBM15 in AMI in vivo

As presented in Figure S2A, sh-RBM15 was successfully transfected into AC16 cells. Then the effect of RBM15 knockdown on AMI in vivo was explored. Western blot assay showed that RBM15, ACSL4 and NCOA4 protein levels were increased and GPX4 and FIH1 protein levels were reduced in the tissues of MI groups, while the effects were abated in MI + sh-RBM15 group (Figure S2B). The pathological score in MI was group was increased, while it was restored in MI + sh-RBM15 group (Figure S2C).

## Discussion

Studies have shown that oxidative stress, lipid peroxidation, inflammation and mitochondrial dysfunction lead to myocardial cell loss and death, which are involved in myocardial I/R injury [23–26]. In recent years, more and more studies have found that there is a new form of cell death, namely ferroptosis, in the pathological process of myocardial I/R injury [27]. In the myocardial tissues of AMI patients, there are pathological changes closely related to ferroptosis, such as iron metabolism disorder, lipid peroxidation and increased ROS [28–30]. In this study, the cell model of myocardial H/R injury was established by treating AC16 cells with H/R and then the effect of RBM15 and ACSL4 on the ferroptosis in H/R-induced AC16 cells was explored. It was demonstrated that RBM15/ACSL4 axis aggravated H/R-induced AC16 cell injury by inducing ferroptosis.

m6A RNA methylation regulators have been confirmed to be involved in the occurrence and development of a variety of diseases [31, 32]. However, their role in AMI has not been fully elucidated. In our present research, RBM5 mRNA level was elevated in AMI patients. cTnI and CK-MB can be used as the markers of myocardial injury and an important reference index for AMI prognosis [33]. The results of this study showed that cTnI and CK-MB levels were positively correlated with RBM15 level in AMI patients. This finding suggested that RBM15 could be a prognosis marker in AMI.

Subsequently, we explored the exact roles of RBM15 in H/R-induced AC16 cell injury. As a result, RBM15 silencing promoted the viability of H/R-induced AC16 cells. Ferroptosis is a core remodeling event after myocardial I/R injury and involves Fe^2+^ overload, intracellular GSH ablation, and reduced GPX4 activity, which leads to ROS generation and lipid peroxidation [34, 35]. RBM15 deficiency induced ferroptosis in lung cancer through altering TGF-β/Smad2 pathway [36]. RBM15 suppressed the ferroptosis and promoted proliferation and metastasis in osteosarcoma cells by mediating the m6A modification of MAT2A [37]. RBM15 stabilized XPR1 mRNA through m6A modification to regulate lung adenocarcinoma cell proliferation, invasion, apoptosis, oxidative stress and ferroptosis [38]. However, the role of RBM15 in H/R-induced cardiomyocyte cell ferroptosis is not reported. Our results indicated that RBM15 knockdown reduced LDH intensity, ROS generation, MDA content, Fe^2+^ level, ACSL4 level, NCOA4 level and GSSG content and elevated SOD activity, GPX4 level, FTH1 level, GSH level and GSH/GSSG ratio in H/R-treated AC16 cells. These outcomes suggested that RBM15 knockdown inhibited H/R-induced ferroptosis in human cardiomyocyte cells.

Afterward, RBM15 was demonstrated to regulated ASCL4 expression through mediating m6A modification of ACSL4. ACSL4 is a kind of lipid metabolic enzyme and severs as a biomarker of ferroptosis due to its contribution to the accumulation of lipid intermediate [39]. Therefore, ACSL4 is an important pharmacological target for the treatment of ferroptosis-related diseases [40, 41]. Zhou et al. suggested that ACSL4 could be targeted by ALKBH5 via m6A modification to alter ferroptosis in hyperbilirubinemia-induced brain damage [42]. Fan et al. verified that Baicalin prevented myocardial I/R injury by repressing ACSL4 expression [43]. In this paper, we firstly investigated the relation between RBM15 and ACSL4 in regulating H/R-induced damage of cardiomyocyte cells. As a result, ACSL4 upregulation restored the effects of RBM15 knockdown on H/R-induced AC16 cell ferroptosis.

Taken together, RBM15 stabilized and regulated ACSL4 through m6A modification, aggravating H/R-induced AC16 cell ferroptosis. Our study provided a novel axis of RBM15/ACSL4 in regulating H/R-induced cardiomyocyte cell damage. Moreover, the study might give a reference for the treatment of myocardial ischemia-reperfusion (I/R) injury through inhibiting ferroptosis.

## Electronic supplementary material

Below is the link to the electronic supplementary material.

Supplementary Material 1

Supplementary Material 2: Figure S1. ACSL4 knockdown repressed H/R-induced ferroptosis in AC16 cells. (A) ACSL4 protein level in AC16 cells transfected with si-NC or si-ACSL4 was measured by western blot. (B-E) After AC16 cells were treated with H/R, H/R+si-NC or H/R+si-ACSL4, the levels of Fe2+, GSH, GSSG and GSH/GSSG in AC16 cells were examined with indicated commercial kits. **P<0.01, ***P<0.001.

Supplementary Material 3: Figure S2. Function of RBM15 in AMI in vivo. (A) RBM15 protein level in sh-RBM15 or sh-NC transfected AC16 cells was measured by western blot. (B) Western blot assay was used to measure the protein levels of RBM15, ACSL4, GPX4, FIH1 and NCOA4 in myocardial tissues in Sham, MI and MI+sh-RBM15 groups. (C) The pathological score in each group was determined. **P<0.01, ***P<0.001.

## Data Availability

No datasets were generated or analysed during the current study.
