# Supplementary material for: RBM15 promotes hypoxia/reoxygenation-induced ferroptosis in human cardiomyocytes by mediating m6A modification of ACSL4
Source: Hereditas. 2025 Jul 18;162:135. doi: 10.1186/s41065-025-00453-0 (PMC12273425; doi:10.1186/s41065-025-00453-0)

Fig.2B

RBM15
(107kDa)

β-actin
(42kDa)

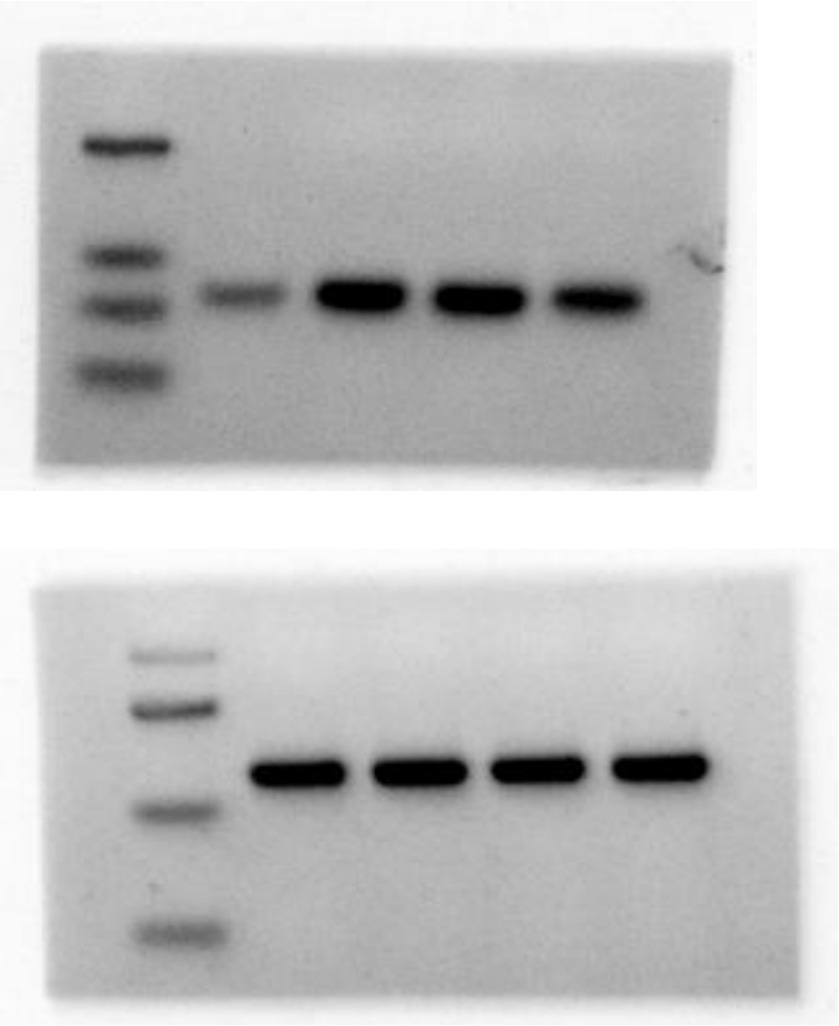

Fig.3B

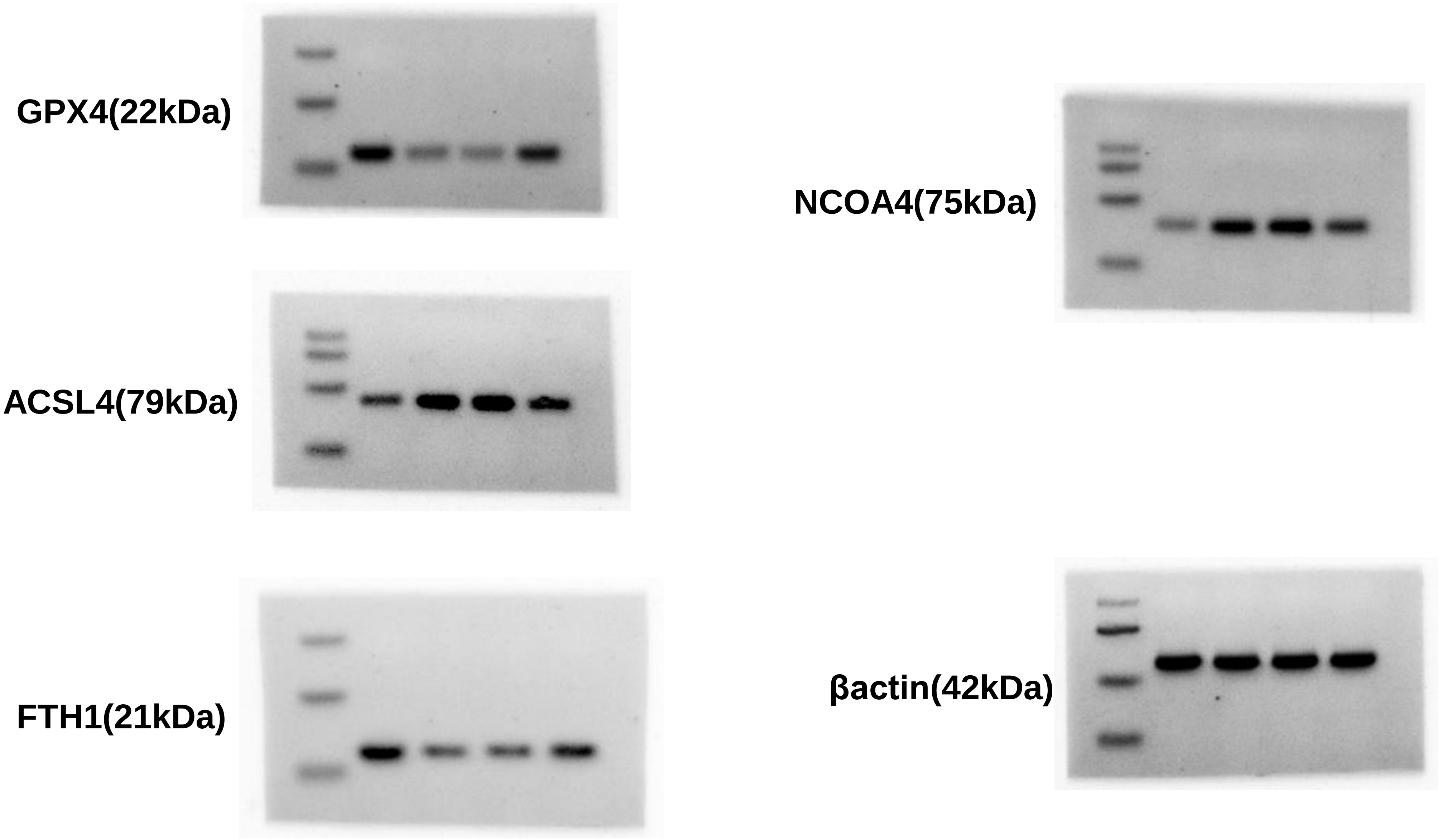

Fig.5A

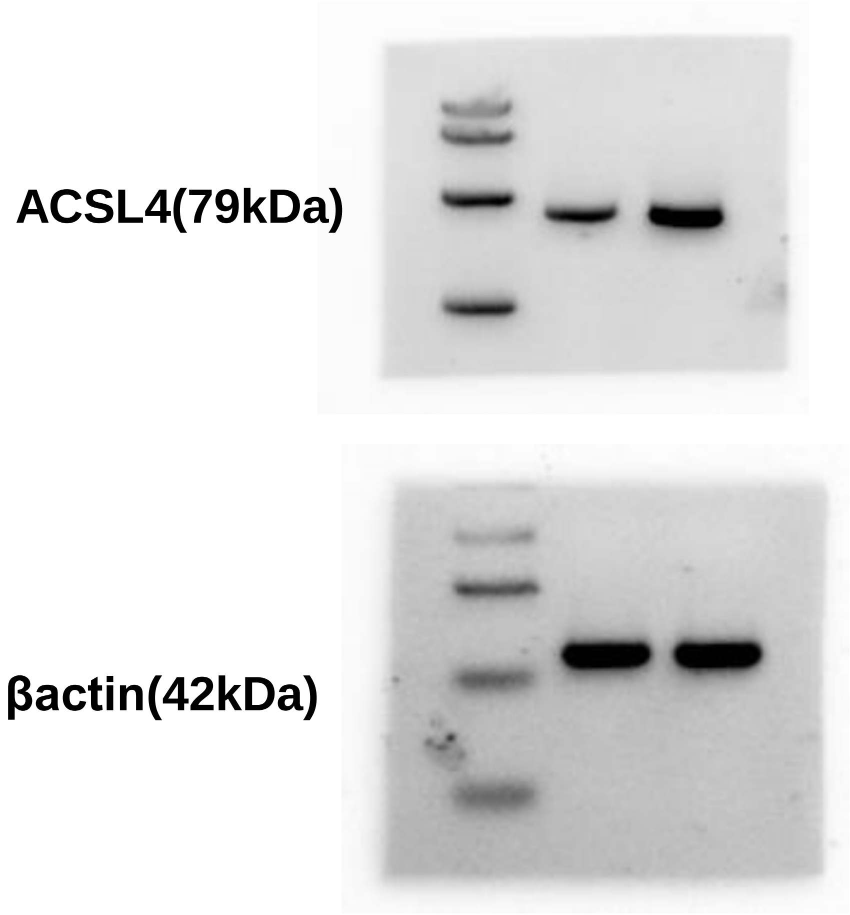

Fig.5H

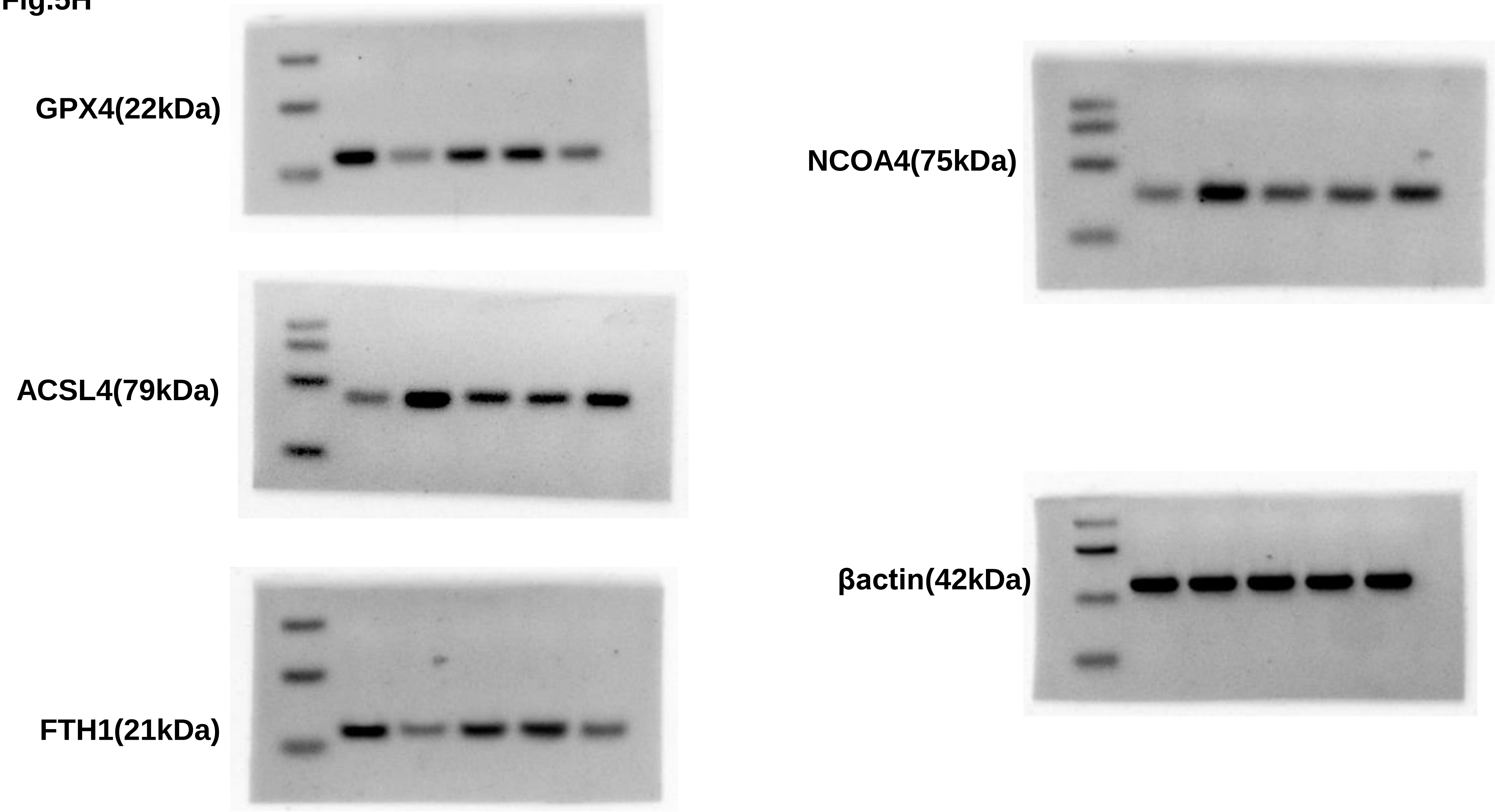

marker

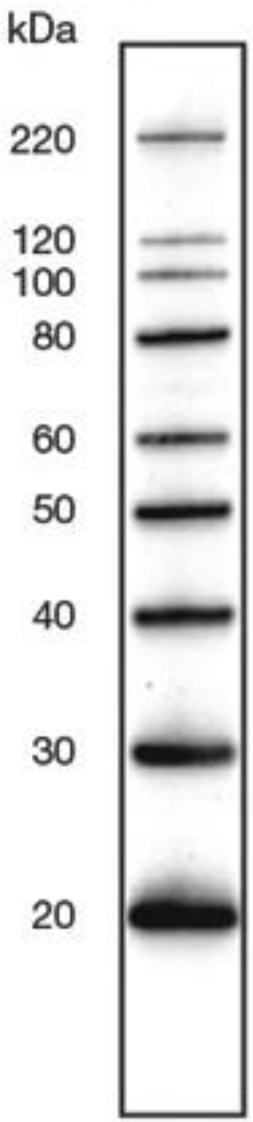

Supplement: Supplementary file 1 — Supplementary Material 1 [file 41065_2025_453_MOESM1_ESM.pdf]
