# Supplementary figures and images for: RBM15 promotes hypoxia/reoxygenation-induced ferroptosis in human cardiomyocytes by mediating m6A modification of ACSL4
Source: Hereditas. 2025 Jul 18;162:135. doi: 10.1186/s41065-025-00453-0 (PMC12273425; doi:10.1186/s41065-025-00453-0)

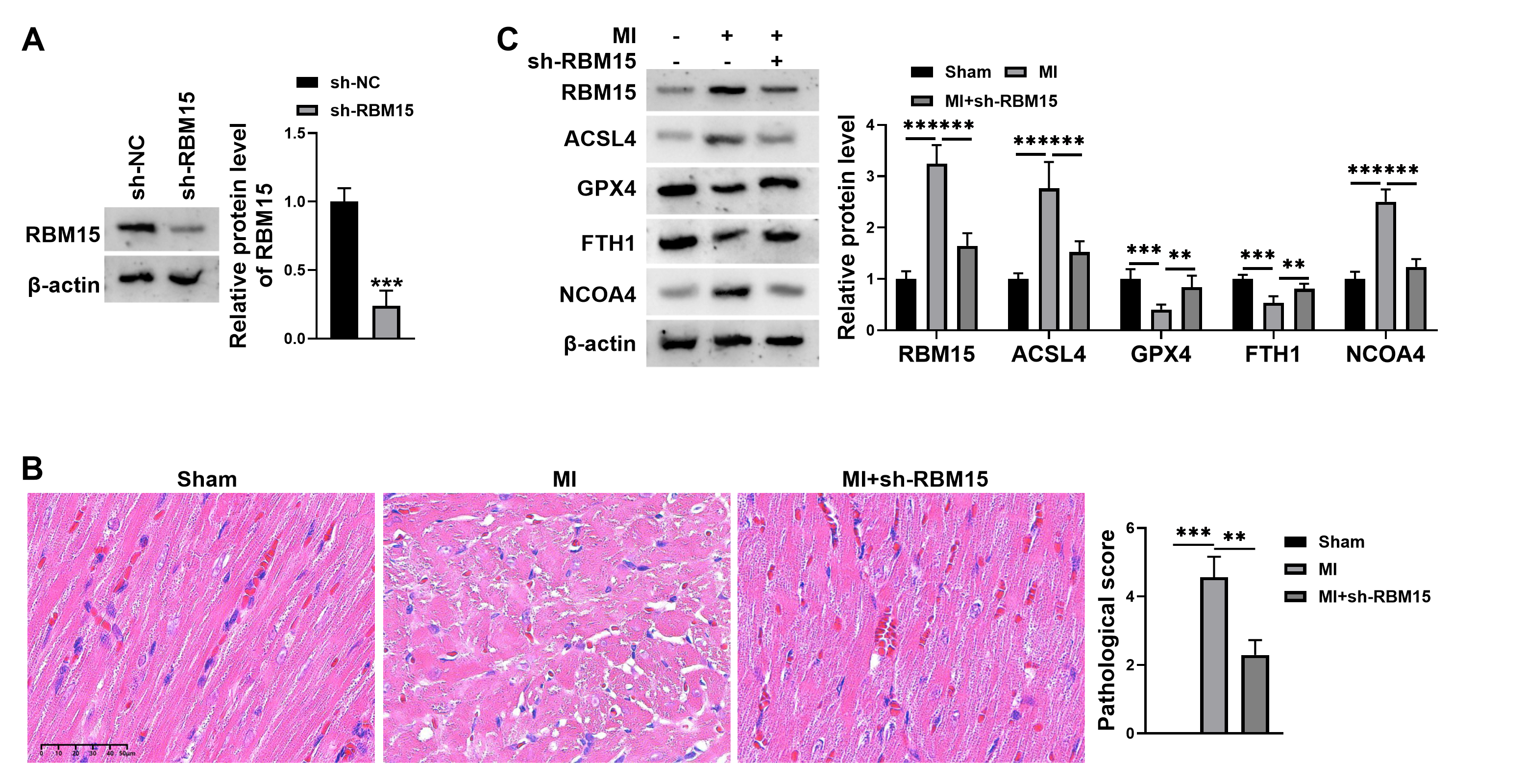

Supplement: Supplementary file 2 — Supplementary Material 2: Figure S1. ACSL4 knockdown repressed H/R-induced ferroptosis in AC16 cells. (A) ACSL4 protein level in AC16 cells transfected with si-NC or si-ACSL4 was measured by western blot. (B-E) After AC16 cells were treated with H/R, H/R+si-NC or H/R+si-ACSL4, the levels of Fe2+, GSH, GSSG and GSH/GSSG in AC16 cells were examined with indicated commercial kits. **P<0.01, ***P<0.001. [file 41065_2025_453_MOESM2_ESM.tif]

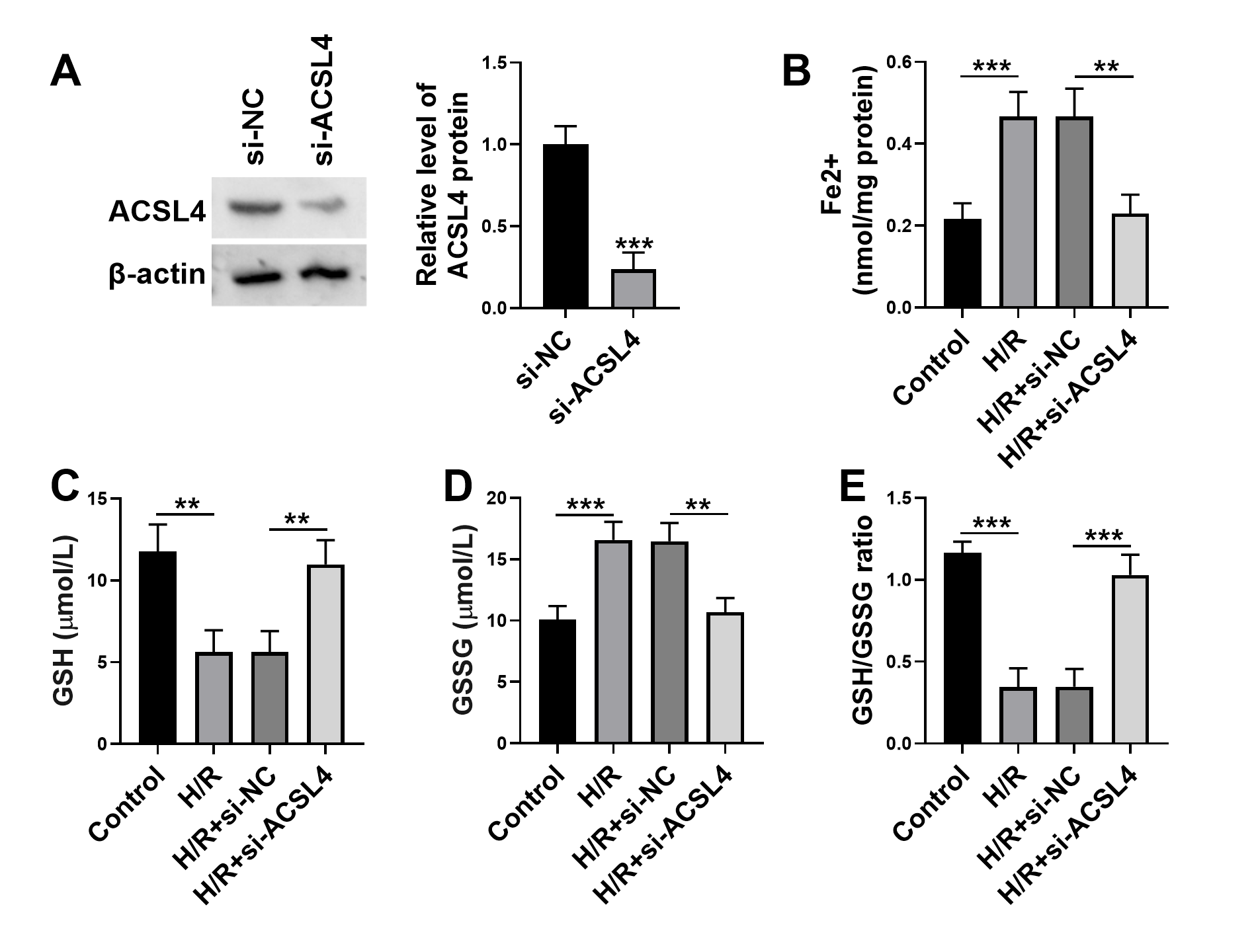

Supplement: Supplementary file 3 — Supplementary Material 3: Figure S2. Function of RBM15 in AMI in vivo. (A) RBM15 protein level in sh-RBM15 or sh-NC transfected AC16 cells was measured by western blot. (B) Western blot assay was used to measure the protein levels of RBM15, ACSL4, GPX4, FIH1 and NCOA4 in myocardial tissues in Sham, MI and MI+sh-RBM15 groups. (C) The pathological score in each group was determined. **P<0.01, ***P<0.001. [file 41065_2025_453_MOESM3_ESM.tif]
